# Phrase-level emotional salience modulates neural substrates of situation model building in developing readers

**DOI:** 10.1016/j.dcn.2026.101707

**Published:** 2026-03-13

**Authors:** Andrea N. Burgess, Sarah S. Hughes-Berheim, Laurie E. Cutting

**Affiliations:** a Peabody College, Vanderbilt University, Nashville, TN, United States; b Vanderbilt Brain Institute, Vanderbilt University, Nashville, TN, United States

**Keywords:** Arousal, Reading comprehension, Functional MRI (fMRI), Situation model, Discourse processing, Emotion-cognition interaction, Child development

## Abstract

Existing neurocognitive reading models highlight a left-lateralized brain network supporting word- to discourse-level processing, but they largely overlook emotion. Although emotion-related brain regions are active during discourse processing, the role of arousal (i.e., emotional intensity) remains underexplored. Prior neuroimaging work has shown that isolated words or whole passages varying in arousal evoke activity in brain regions associated with emotion and situation model processing. However, how arousal at the phrase level within passages may modulate neural activity is unclear, nor have studies investigated how individual differences in arousal responsiveness may be linked to reading comprehension ability, particularly in developing readers. Here, we used functional magnetic resonance imaging (fMRI) to examine the neural correlates of lexical arousal in 86 third-graders as they read passages. A parametric modulation analysis, using phrase-level arousal ratings from a validated lexical database, was used to investigate how fluctuations in arousal during passage reading correlated with neural activity. We demonstrated that phrase-level arousal was associated with increased activity in regions implicated in emotional processing and situation model construction: the right amygdala, striatum, and posterior insula, and the left dorsomedial prefrontal cortex (dmPFC). Additionally, dmPFC activity was associated with better reading comprehension ability, aligning with prior literature linking dmPFC to situation model building. This work highlights the importance of integrating lexical emotional dimensions into cognitive models of reading and supports the idea of using emotionally engaging materials to enhance comprehension for developing readers of all abilities.

## 1 Introduction

The ability to read is a skill that has far-reaching implications both in and out of the classroom. Reading competence is crucial for long-term educational attainment, social functioning, and health outcomes (DeWalt et al., 2004, Pace et al., 2019). Despite concerted efforts from practitioners, researchers, and policymakers to improve children’s reading abilities, a majority of students in the United States still struggle to reach a proficient status in reading (NCES, 2025). Learning to read requires the coordination of numerous neural systems (Martin et al., 2015, Murphy et al., 2019, Turkeltaub et al., 2002, Walenski et al., 2019, Yang et al., 2019) that are influenced by multidimensional textual factors (e.g., Barth et al., 2014; Kulesz et al., 2016; McNamara et al., 2011; Tortorelli et al., 2020). One such text characteristic that influences reading comprehension is lexical arousal (Hsu et al., 2015, Pickren et al., 2022), or the intensity of emotion conveyed by a stimulus (Bradley and Lang, 1999; Russell, 1980). However, little is known about the underlying neural functioning associated with arousal responsiveness during discourse processing. Of particular interest is understanding how emotional salience at the word level can impact higher-level processing, especially in regions linked to situation model formation that are thought to be central for proficient comprehension. Understanding these processes is important to inform future interventions, particularly for those before fourth grade, as this marks a developmental period where struggling readers begin to fall behind in reading and other academic subjects that rely on proficient comprehension (Chall and Jacobs, 2003, Wanzek et al., 2010).

Cognitive models of reading have long theorized that reading is a complex process that involves the integration of relevant background knowledge with specific text-based information to form a coherent mental text representation, or *situation model* (Schmalhofer and Glavanov, 1986, van Dijk and Kintsch, 1983). Understanding factors that influence situation model formation, including word-level emotional salience within passages, may be particularly advantageous for providing insights into how to bolster developing readers’ comprehension abilities (e.g., by using manipulation of lexical arousal to improve comprehension). Here, we examined whether fluctuations in arousal at the phrase level during passage reading modulated neural activity in situation model regions in developing readers. Critically, we also investigated whether this neural activity was linked to indices of children’s ability to form situation models during reading (i.e., reading comprehension ability) both in and out of the scanner. Finally, we explored whether this effect varied by children’s decoding level, or their ability to sound out words, to determine whether individual differences in children’s basic reading skills impacted the relationship between text arousal and comprehension.

### 1.1 Cognitive models of reading and affect processing

Cognitive models of reading have shown that different reader characteristics influence the depth and ease with which students form a coherent situation model from texts. While there are many models of reading, both the Construction-Integration Model (Kintsch, 1988) and Reading Systems Framework (Perfetti, 1999) highlight the importance of considering bottom-up (i.e., text-based) and top-down (i.e., reader-specific) features in the reading trajectory. This interaction between bottom-up and top-down processes may be especially important for understanding how a local-level manipulation of arousal may serve to enhance situation model formation during downstream processing. Though the literature on the relationship between arousal and reading ability is still growing, there is strong theoretical backing to support this association. Scholars have conceptualized general stimulus emotionality across three main dimensions: 1) arousal, the activation experienced in response to a stimulus, 2) valence, the pleasantness of a stimulus, and 3) dominance, the control exerted by a stimulus (Bradley and Lang, 1999; Russell and Mehrabian; 1977; Schlosberg, 1954; Warriner et al., 2013). Various researchers have asserted that more emotional texts contribute to stronger situation model development by cluing readers into character and writer motivations (and thus, causal text structure; de Vega et al., 1996), tapping into embodied cognition (Louwerse, 2011, Niedenthal, 2007), and increasing reader attention and engagement (Kneepkens and Zwaan, 1995). Additionally, the Neurocognitive Poetics Model of Literary Reading by Jacobs, 2011, Jacobs, 2014, Jacobs, 2015 is a model that pulls from cognitive, neuroscientific, literary, and aesthetic fields to assess how various text features, such as emotional affect, interact to give rise to a reader’s coherent text percept. The theory argues that diverse text properties, including emotionality, need to be quantitatively evaluated to understand the cognitive and neuroscientific underpinnings of emotional processing of individual words and, ultimately, passages.

While there is a theoretical basis for the role of emotion in reading comprehension, empirical findings are relatively limited on the topic. There is support from the word-level literature for the impact of arousal on reading. Positive words (e.g., Kazanas and Altarriba, 2016) and more exciting words (e.g., Kever et al., 2019; Recio et al., 2014) tend to be recognized and processed more quickly than neutral words. Additionally, a study by Citron and colleagues (2014) revealed that in a highly controlled lexical decision task, latencies for positive high-arousal and negative low-arousal words were shorter than for positive low-arousal and negative high-arousal words. At the passage level, a few studies have shown that textual emotional content facilitates comprehension in various ways (de Vega et al., 1997, Gernsbacher et al., 1992, Gillioz et al., 2012, Kneepkens and Zwaan, 1995, Pickren et al., 2022). For example, de Vega, Díaz, and León (1997) demonstrated that readers are more sensitive to passage information in texts with higher emotional content. One study by Pickren, Stacy, and colleagues (2022) evaluated whether emotional word-level metrics *within passages* had an impact on reading comprehension in children. In this cohort of 8- to 11-year-old readers, the authors found that, over and above children’s component reading skills and additional text characteristics, lexical ratings of arousal of individual words in the passages contributed to enhanced passage comprehension. These passage- and word-level influences bring into question whether local-level arousal manipulation (i.e., at the level of words or phrases) influences situation model formation and whether these manipulations help readers of different abilities. Clarifying whether local-level arousal manipulation plays a role in situation model construction using neurocognitive approaches is particularly important for understanding how developing readers engage with text, as emotional cues at the local level may offer a pathway to improve comprehension and intervention.

### 1.2 Neural circuitry of reading and affect processing

Reading involves the complex coordination of various brain regions critical for language, vision, memory, emotion, and executive functioning (Bailey et al., 2018, Landi et al., 2013, Schlaggar and McCandliss, 2007). Meta-analytic results reveal that both adults and children show reading-related brain activity in left ventral occipitotemporal (OT), inferior frontal, and posterior parietal regions when processing individual words (Martin et al., 2015, Murphy et al., 2019, Turkeltaub et al., 2002). These “core” reading regions interface with a host of others, as reading moves from word processing, to phrase, to sentence, and finally to discourse processing (Walenski et al., 2019, Yang et al., 2019), allowing proficient readers to develop a coherent mental representation of the text (van Dijk and Kintsch, 1983). At the discourse level, in addition to traditional reading regions linked to word-level processing, networks associated with social cognition and cognitive control are widely engaged (Aboud et al., 2019, Kim et al., 2022, Wang et al., 2019), as well as networks related to emotion processing (Twait et al., 2018). In general, arousing and salient information is processed in a dispersed network of cortical and subcortical structures, including the medial and lateral prefrontal cortex (PFC), cingulate cortex, insula, basal ganglia, hippocampus, and amygdala (Dolan, 2002, Phillips et al., 2003). Of these emotion processing regions, empirical and meta-analytic results suggest that the medial PFC and, to some extent, the amygdala, are involved in situation model formation during reading. Broadly, both the medial PFC and the amygdala have been implicated in discourse processing (Ferstl et al., 2008, Yang et al., 2019), though the amygdala to a lesser extent. The medial PFC has been consistently shown to be active during discourse tasks of inference, coherence (Siebörger et al., 2007) and perspective-taking (Fletcher et al., 1995; Jacoby et al., 2020). Additionally, the medial PFC is a central hub for the default mode network (DMN; Raichle, 2015). Most neurocognitive studies of discourse processing show DMN activation (e.g., Aboud et al., 2016; Aboud et al., 2019; Jacobs and Willems, 2018; Kim et al., 2022; Tylén et al., 2015), typically interpreted as reflecting the formation of an appropriate internal situation model (Buckner et al., 2008, Mar, 2011, Simony et al., 2016). Given the functional overlap in these regions for emotion processing and situation model formation, they serve as strong candidates for investigating how arousal may impact reading comprehension.

Few neurocognitive studies have directly examined whether modulation of emotion during passage reading influences medial PFC or amygdala activity. The existing neurocognitive discourse processing studies that have examined the role of textual arousal in discourse processing have shown that arousal is linked to activity in regions that support emotion, situation model building, and theory of mind processes (Ferstl et al., 2005, Hsu et al., 2015, Wallentin et al., 2011). One of the first studies to investigate how passage-level emotional content influences neural activity was conducted by Ferstl and colleagues (2005). Participants listened to 32, 40-second short stories that contained either emotional or neutral content. The presence of emotional content elicited activation of the ventromedial PFC and amygdala. A similar study of participants listening to a recording of “The Ugly Duckling” reported that sentence-specific arousal ratings corresponded with BOLD activity in temporal and frontal cortices, the thalamus, and the right amygdala (Wallentin et al., 2011). Neither study examined whether these activation patterns were modulated by reader ability, or how well the texts read in the scanner were comprehended. While these studies advanced our understanding of arousal responsiveness within text, they are potentially confounded by the vocal intonation conveyed by the speaker. Having participants *read* passages, as Hsu et al. (2015) did, may more accurately reveal the association between affect and neural discourse circuitry. Hsu and colleagues measured passage arousal in two ways, either indexed by the adult participants’ ratings of the passages or by average arousal metrics of the constituent words determined by independent norms (Schmidtke and Conrad, 2018) and found that both quantifications modulated the aforementioned neural systems. Most interestingly, they found that the range of arousal ratings across a passage modulated activity in the left amygdala and insula, among other attention, situation model building, and theory of mind regions. These findings suggest that manipulation of affect in texts impacts situation model processing. However, because reader-specific comprehension metrics were not included as a factor in the study, their relationship with comprehension and situation model building remains unknown.

### 1.3 Current study

To date, despite the contribution of the aforementioned literature, neuroscientific studies have only been conducted in adults; have only examined arousal at the passage level; have not linked neural activation to reading comprehension metrics (both of the specific in-scanner passages read, as well reading comprehension more generally); and have not examined whether reader (decoding) ability impacts findings. As such, whether arousal modulation of the neural situation model regions occurs at the global level (passage) or local level (word or phrase) is unclear. Further, it is unknown whether these arousal fluctuations are linked to out-of-scanner or in-scanner metrics of reading comprehension ability, or if these relationships vary by reader ability. There is support from the literature on word-level processing to suggest that local phrase-level manipulation of arousal during discourse processing could result in modulation of emotion-related and situation model regions. Indeed, across various paradigms, emotionally laden words have been found to activate the striatum (Hamann and Mao, 2002, Lewis et al., 2007), PFC (Compton et al., 2003, Posner et al., 2009, Straube et al., 2011), cingulate cortex (Compton et al., 2003, Lewis et al., 2007, Posner et al., 2009), insula (Citron and Gray, 2011, Lewis et al., 2007), and amygdala (Hamann and Mao, 2002, Lewis et al., 2007, Straube et al., 2011), amongst other regions (for a review, see Citron, 2012). Therefore, there is reason to believe that manipulation of arousal at the local level may have an impact on situation model processes, particularly for those readers with weaker word-level (decoding) skills. Understanding whether this is true is particularly relevant for outlining how developing readers process text, as manipulation of emotion at the local level could contribute to enhanced comprehension.

Here, we examined whether a local-level metric of arousal, using standardized lexical norms, fluctuated in time with activity in situation model or emotion-related brain regions in 86 third-graders with varying reading abilities. We expected arousal metrics to modulate regions associated with emotional processing, including the amygdala, insula, cingulate cortex, and medial PFC; however, we additionally hypothesized that among those regions, only those linked to situation model processing (i.e., medial PFC and potentially amygdala) would be linked to in-scanner comprehension of the specific passages read, as well as to an out-of-scanner general reading comprehension measure. Finally, we hypothesized that reader decoding ability would interact with neural fluctuations during reading and potentially provide an enhanced benefit for weaker compared to stronger readers.

## 2 Methods

### 2.1 Participants

Participants were drawn from a larger longitudinal study tracking reading development in a cohort of elementary-aged students. Recruitment occurred in public and private schools, homeschool networks, community centers, and pediatric offices, as well as on social media, in the greater Nashville and Middle Tennessee area in the Southeastern United States. Participants were American English speakers with typical or corrected visual and/or hearing differences and no known history of pervasive developmental disorders, including severe language disabilities, major psychiatric or neurological diagnoses, or contraindications for MRI scanning. All study procedures were carried out under the university’s internal Institutional Review Board (IRB). Written parental or guardian consent was obtained prior to the start of data collection, and children’s assent was monitored throughout study participation. All participants were compensated for their study involvement.

During the summer and early fall following the successful completion of third grade, 149 children were brought in for data collection. Of those children, 105 participants opted into MRI scanning, and 86 of those participants were included in the final analysis after data cleaning (see *2.8 fMRI data cleaning* below). Demographics were reported by the child’s parent or legal guardian. This sample (*M* age = 9.44 ± 0.39 years) included 44 girls and 42 boys, with 1.2% of the sample identifying as Asian (1 child), 8.1% as Black (7 children), 79.1% as White (68 children), 9.3% as more than one race (8 children), and 0% as Alaskan Native, Native American, Pacific Islander, or Native Hawaiian; 2.3% (2 children) preferred not to report. Additionally, 5.8% of the sample (5 children) was reported to identify as Hispanic/Latine, and 2.3% (2 children) preferred not to report. The Hollingshead (1975) system was used to compute family socioeconomic status (SES), based on individual or couple education and occupation. Parent or legal guardian household scores on the assessment can range from 8 to 66. Our particular sample had scores ranging from 36 to 62, which correspond to social-class descriptors of middle to upper class. Most parents received an educational score of six, indicating they had completed a bachelor’s degree.

### 2.2 Behavioral testing

Children participated in comprehensive behavioral testing during their visits. All participants were confirmed to have typical Full Scale IQ scores (standard score > 75 on the Wechsler Abbreviated Scale of Intelligence (WASI) Second Edition; Wechsler, 2011), as intellectual disability may act as a confound for studying reading variability in the brain (Nilsson et al., 2021). Children also completed subtests from the Woodcock-Johnson IV Tests of Achievement (WJ): the Letter-Word Identification and Word Attack subtests, and the Passage Comprehension (PC) subtest from the Tests of Oral Language (Schrank and Wendling, 2018). Sample descriptive statistics are reported in Table 1. Mean scores indicated that the sample performed within the average range. Range scores highlight the variability of the sample, indicating that participants fell within the full range of reading ability, from low to high performing readers. Missing values for subsequent analyses using behavioral measures were mean replaced.

**Table 1:** Descriptive statistics for *n* = 86

Measure	Mean (SD)	Range	Percentile
Age (years)	9.44 (0.39)	8.33 – 10.33	
SES	53.76 (6.44)	36 – 62	
WASI Full Scale IQ *	106.99 (14.50)	79 – 136	8 – 99th %
WJ Letter-Word Identification *	108.71(10.92)	90 – 132	25 – 98th %
WJ Word Attack*	107.82 (11.94)	78 – 132	7 – 98th %
WJ Passage Comprehension *	103.46 (11.85)	78 – 131	7 – 98th %

Table 1. Age, SES, behavioral IQ, and reading scores for the current sample of third-grade students. Note: SES = socioeconomic status couple score; * = Age-corrected standard scores.

### 2.3 Stimuli

#### 2.3.1 Passages

During each scanning session, participants completed structural and functional sequences, including resting state, listening comprehension tasks, and two *Passage Reading* tasks used for the current study. These passages consisted of one passage that was about funguses and another that was a fable about popularity, henceforth referred to as Passage 1 and Passage 2, respectively. Passages were constructed in-lab and were equated across the following Coh-Metrix metrics (McNamara et al., 2014): word count, sentence length, word length, CELEX word frequency, word concreteness, Flesch Reading Ease, and Flesch-Kincaid Grade Level (Table 2). To equate the passages, we utilized an iterative leave-one-out bootstrapping procedure across all experimental passages (which included 10 in-scanner passages and 10 out-of-scanner passages from across the longitudinal study). During this process, one passage was left out at a time, and then the 95% confidence interval for each metric was calculated (less word count and Flesch-Kincaid Grade Level). If passage means fell within the confidence intervals of the other passages, they were considered equated. This procedure was replicated for each functional MRI passage.

**Table 2:** Average passage statistics

Measure	Passage 1	Passage 2
Word Count	150	150
Sentence length	10.79	10.71
Word length	1.35	1.36
CELEX word frequency	2.31	2.28
Word concreteness	418.75	429.86
Flesch Reading Ease	81.59	80.90
Flesch-Kincaid Grade Level	4.56	4.64

Table 2. Descriptive statistics for Passage 1 and Passage 2, as derived from Coh-Metrix.

#### 2.3.2 Experimental design

Passage 1 and Passage 2 tasks were completed in separate functional runs, with passage order counterbalanced across participants. We employed a combined block and event-related design. Each functional run contained 3 conditions (Passage Reading, Scrambled Phrases, and Fixation) across 12 blocks (4 blocks per condition). Scrambled Phrases were matched in content and function words to the coherent Passage Reading condition, but words were randomized within each “idea unit” (see 2.3.3. *Stimuli presentation* below). As we were interested in investigating situation model processing, including both word reading and discourse construction processes, the Scrambled Phrases baseline condition was not included in our current analysis. Blocks of Fixation of approximately 23 s were interspersed throughout the task and used as the baseline condition for this analysis. The total runtime for each functional run was 7 min and 31 s, and within a functional run, each condition (without jitter) ran for approximately 91.5 s across 4 blocks.

#### 2.3.3 Stimuli presentation

We presented passages as “idea units”, an established approach that we have used in prior studies (Aboud et al., 2016, Aboud et al., 2019, Kim et al., 2022, Swett et al., 2013, Wang et al., 2019), mimicking the pace of natural reading while reducing eye movements. As in Swett et al., 2013, these idea units were short snippets of text (1–5 words in length) that followed the grammatical and intonational breaks of the passage and included noun phrases, verb phrases, and prepositional phrases. To determine the idea units, at least two lab members individually created idea units based on the grammatical and intonational breaks they saw fit. When disagreements occurred, a third person was involved to resolve the discrepancies. All final idea units were agreed upon by all lab members.

**Fig. 1:**
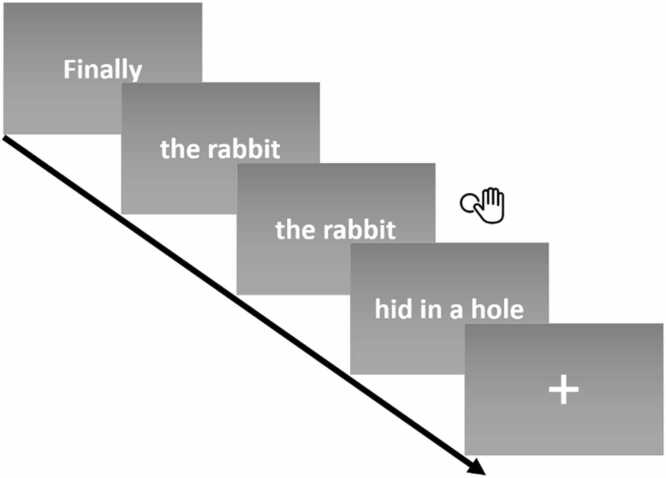
Sample stimuli presentation. As an attention check, children were asked to indicate consecutively repeated stimuli using a thumb button press. Not pictured is the blank screen (variable jitter) between each stimulus.

We allowed 550 ms for each word in the trial (modified from Aboud et al., 2016; Swett et al., 2013). For example, a trial with 3 words would be presented for 1650 ms. Between each stimulus, a blank screen with a randomized jitter between 250 and 2600 ms was displayed to allow for the appropriate modeling of event-related BOLD activity (Henson, 2007). See Fig. 1 for an example trial.

### 2.4 Quantifying phrase arousal

**Fig. 2:**
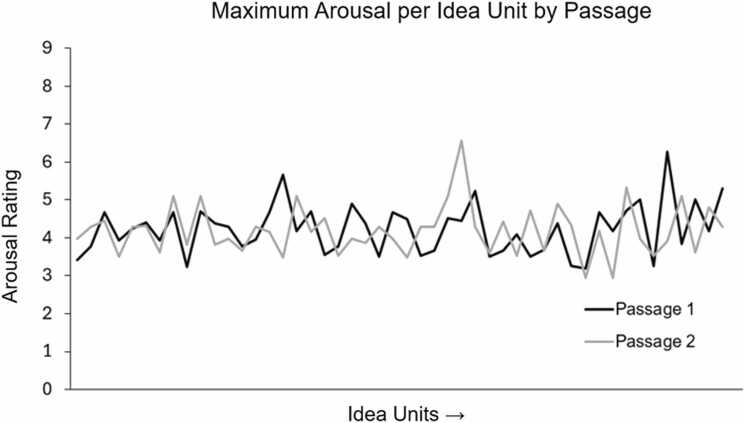
Plot of the maximum arousal rating for each idea unit, after removing unrated idea units. Black = Passage 1, Grey = Passage 2.

To quantify the lexical arousal of each idea unit, we leveraged the database of affective ratings for well-known English content words collected by Warriner and team (2013). Approximately 1800 respondents contributed to this dataset, rating (on a 9-point Likert scale) nearly 14,000 English lemmas on the dimension of arousal, among other metrics. Here, higher arousal ratings indicated that participants felt excited, stimulated, or frenzied by the word, while lower arousal ratings indicated that participants felt calmed, relaxed, or dulled by the word. These norms have been used prolifically and validated across fields and in a host of research settings (e.g., Dong et al., 2024; Dong and Nation, 2025; Sinclair et al., 2021). Since the rating database contained only lemmas, we transformed all content words into their singular, non-possessive, present tense forms. Passages contained between 2 and 4 content words that did not have an arousal rating; these words were included, but values were marked as missing. After each content word was rated, the highest arousal rating in each idea unit was assigned as the arousal rating for that trial for modeling. Arousal ratings were mean centered across all conditions. Idea units that had no content words, and thus no arousal rating per trial, were regressed out in the first-level analysis. Passage 1 and Passage 2 had 15.5% unrated idea units each. Passage 1 (*M* = 4.23 ± 0.45, range = 3.19–6.26) and Passage 2 (*M* = 4.19 ± 0.43, range = 2.94–6.57) did not differ by idea unit maximum arousal rating (*t*_96_ = 0.28, *p* = .78; two-sample *t*-test). See Fig. 2 for a plot of the fluctuations in arousal ratings by passage.

### 2.5 In-scanner behavior

We utilized a multistep procedure to acclimate children to scanning, including a play tunnel procedure, a mock scanner with scanner sounds, and task practice with separate stimuli. During the actual scan session, a mounted mirror on the head coil allowed participants to view stimuli projected onto a screen in the scanning room. While reading, to gauge in-scanner attention, participants were asked to monitor for randomly repeated stimuli on two successive screens. Children indicated these repetitions with a right thumb press on a button box. During each functional run, 8% of the stimuli were consecutively repeated. Repetitions were treated as regressors of no interest in the first-level analysis. Although passages were purposefully chosen to be about obscure topics, children were asked if they had any knowledge of the passage topics prior to scanning to control for any background knowledge that may have influenced comprehension, thus allowing for determining the what participants learned from the passages (from here on we refer to this calculation as *Amount Learned* to signify while it is a comprehension measure, it accounts for background knowledge). Their verbal responses were coded and compared against a checklist of passage idea units to assess overlapping background knowledge. Immediately following the individual scans, the in-scanner participants were asked to freely recall the passages: “Please tell me everything you read about [The Popular Hare/Funguses].” Again, their responses were coded and compared against passage idea units to assess overlap. The number of idea units recalled was recorded as their recall score. The amount a participant learned from each passage was then calculated by subtracting their background knowledge score from the total number of idea units recalled after reading each in-scanner passage. *Amount learned* across each in-scanner passage was summed and this variable was included as a measure of reading comprehension in subsequent analyses.

To ensure that participants had attended to the in-scanner stimuli, we required them to have either: 1) at least 50% correct responding on the repetition trials (responding when they should be) and less than 5% sporadic responding on non-response trials (responding when they should not be), or 2) recall at least a minimal amount of additional passage information (at least one additional idea unit) than was reported prior to scanning. The first criterion was included to gauge basic attention during the task, and the second criterion was included to ensure participants had been engaged in reading the passage. Based on these criteria, 5.4% of the data were excluded (11 functional runs; see *fMRI data cleaning* below).

### 2.6 Image acquisition

All scans were collected on a Philips Achieva 3.0 T MR scanner with a 32-channel head coil at the Vanderbilt University Institute of Imaging Science. Blood oxygen level-dependent (BOLD) neural responses were collected during two runs of the reading task using T2*-weighted echo planar imaging (EPI) sequences. Each functional run lasted for 7 min and 31 s, or 205 dynamics (plus 5 initial discarded dummy volumes). Each dynamic contained 40 interleaved slices oriented in parallel in the transverse plane. Additional scanning parameters included slice thickness = 3.0 mm with no gaps; echo time (TE) = 30 ms; repetition time (TR) = 2200 ms; flip angle = 75°; field of view (FOV) = 240 × 240 × 120 mm (excluding the cerebellum); matrix size = 80 × 80; voxel size = 3 mm^3^. In addition to EPI sequences, standard T1-weighted structural images were also collected and used for co-registration.

### 2.7 fMRI data preprocessing

Functional images were preprocessed and analyzed using Statistical Parametric Mapping 12 (SPM12; Wellcome Trust Centre for Neuroimaging, London, UK; https://www.fil.ion.ucl.ac.uk/spm/; Penny et al., 2011) in MATLAB (R2024a; MathWorks, Natick, MA, US). Participants’ functional images were slice-time corrected, realigned to the average functional volume, co-registered with their individual brain-extracted T1-weighted structural image, normalized to MNI space, and spatially smoothed using a 6-mm FWHM Gaussian filter. Raw and preprocessed images were visually inspected for appropriate spatial fit along axial, sagittal, and coronal planes.

Functional image motion parameters were derived using Artifact Detection Tools (ART; http://www.nitrc.org/projects/artifact_detect/). We calculated motion in the six planes of movement and derived outlying volumes as volumes having a global-signal *z*-value above 5 and/or volume-to-volume motion greater than 0.9 mm. To be included in the current analysis, participants needed to display fewer than 25% outlying volumes. Passage 1 (*M* % outliers = 10.1 ± 0.4) and Passage 2 (*M* % outliers = 8.7 ± 0.4) task runs did not differ on the percentage of identified outlier volumes (*t*_85_ = 1.59, *p* = .12; mean-replaced paired *t*-test).

### 2.8 fMRI data cleaning

At analysis onset, data were available from 105 children with 203 functional runs. As mentioned above, we used visual inspection techniques to assess scan usability. Of the total group, 4 functional runs (2 participants) were deemed to have insufficient head coverage, 6 functional runs (3 participants) had large incidental findings that interfered with the normalization process, and 8 functional runs (4 participants) experienced a processing pipeline failure. We also examined participant in-scanner behavior for data exclusion: 6 functional runs (3 participants) were excluded because the participant fell asleep, 20 functional runs (across 17 participants) were removed for excessive movement, and 11 functional runs (across 8 participants) were excluded for insufficient repeat monitoring or passage recall. Following this cleaning procedure, our resultant group included 149 functional runs across 86 participants; 61 participants had both functional runs usable, and 25 participants had either a Passage 1 or Passage 2 functional run usable.

### 2.9 fMRI data processing

First-level analyses were conducted using SPM12. Event-related general linear models (GLMs) were generated for each participant. Each condition (i.e., Passage Reading, Fixation) was convolved with a canonical hemodynamic response function (HRF). Motion parameters, button press response trials, and trials without an arousal rating were added to the GLMs as regressors of no interest. A contrast of Passage Reading > Fixation (concatenated across both functional runs at the first-level) was generated for each participant. To examine the contributions of idea unit arousal to the BOLD signal fluctuation, we utilized a parametric modulation approach to look at the intercorrelation pattern between arousal ratings and functional activity during passage reading. In other words, we investigated whether the BOLD signal increased or decreased with the intensity of the parameter of interest (i.e., arousal) by weighting the HRF signal for each event with the trial arousal rating. All fMRI findings were threshold-free cluster enhanced (TFCE, 10,000 permutations; Smith and Nichols, 2009), and multiple comparisons were controlled at a false discovery rate (FDR) of *p* < .05. All fMRI models included age, gender, and SES as control variables. Regression models predicting in- and out-of-scanner reading comprehension included these same control variables; Word Attack was also included to examine any interactions between reading abilities and activation in predicting reading comprehension outcomes.

## 3 Results

### 3.1 Effects of Passage Reading > Fixation

**Fig. 3:**
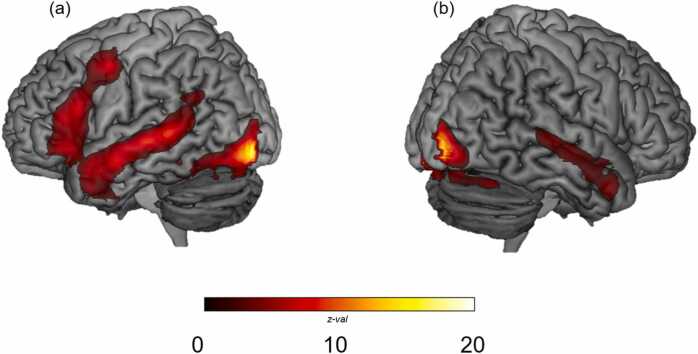
Activity for the GLM Passage Reading > Fixation in the (a) left hemisphere and (b) right hemisphere. We observed recruitment of bilateral reading and language regions. TFCE results displayed at *p* FDR-corrected < .05.

We first examined the recruitment of areas for Passage Reading (both runs) over and above activity related to Fixation (Fig. 3; Table 3). As expected, we observed bilateral activation of canonical reading regions. In the left hemisphere, passage reading recruited increased activation in the anterior temporal lobe and along the length of the superior temporal sulcus, the angular gyrus, medial PFC, inferior frontal gyrus, precentral gyrus, insula, visual cortex, and ventral occipitotemporal cortex (vOTC). Additionally, in the right hemisphere, we observed similar but less widespread activity in homologous regions, including the anterior temporal lobe, superior temporal sulcus, medial PFC, and visual cortex.

**Table 3:** Activations for Passage Reading > Fixation

Cluster size (*k*)	Anatomical region	BA	Max *T*	x	y	z
**14495**	**LH lingual gyrus, cuneus** LH lingual gyrus RH lingual gyrus LH lingual gyrus RH extrastriate cortex RH PVC LH fusiform gyrus LH MTG LH STG LH posterior MTG LH MTG LH STG LH SMA RH middle occipital gyrus LH IFG	**18** 18 18 18 18 17 18 21 22 21 20 21 6 19 45	**21.21** 20.11 19.85 19.77 19.65 15.62 13.57 11.58 11.48 11.27 11.22 11.03 10.90 10.86 10.78	**-15** -27 21 -23 19 13 -36 -49 -53 -61 -55 -55 -49 35 -55	**-92** -94 -90 -92 -92 -86 -86 -46 -38 -30 -10 -24 -2 -86 24	**-8** -8 -6 -8 -2 2 -13 6 4 -2 -14 -4 48 -8 12
**2622**	**LH putamen** LH parahippocampal gyrus RH thalamus RH putamen RH precuneus RH anterior cingulate cortex RH anterior cingulate cortex	**48** 27 20 — 27 25 25	**8.67** 8.46 7.57 5.67 5.49 4.81 4.47	**-22** -21 25 20 7 9 9	**12** -30 -26 8 -36 12 13	**4** -6 -7 2 7 10 8
**1016**	**LH SMA** LH SMA LH dmPFC LH dmPFC LH medial frontal gyrus RH dmPFC RH dmPFC	**6** 6 9 9 8 9 9	**8.43** 7.79 6.77 6.32 5.20 4.70 4.68	**-3** -5 -9 -5 -11 7 7	**4** 6 48 52 30 50 46	**62** 56 44 34 50 32 34
**1891**	**RH STG** RH MTG RH MTG RH temporal pole RH STG RH insular cortex RH temporal pole RH temporal pole	**21** 20 20 20 21 38 38 20	**8.34** 7.24 7.11 7.04 5.96 5.64 5.02 4.75	**53** 51 51 47 49 43 55 37	**-34** -14 -18 8 4 12 10 16	**2** -16 -12 -28 -14 -22 -16 -34

Table 3. Note: Cluster size in mm^3^. TFCE results reported at *p* FDR-corrected < .05. For large clusters, sub-cluster peaks distinct from the primary peak are reported, extracted using a decreased peak search space of 4 mm within the main cluster. BA: Brodmann area, PVC: primary visual cortex, MTG: middle temporal gyrus, STG: superior temporal gyrus, SMA: supplementary motor area, IFG: inferior frontal gyrus, dmPFC: dorsomedial prefrontal cortex.

### 3.2 Effects of arousal during Passage Reading

**Fig. 4:**
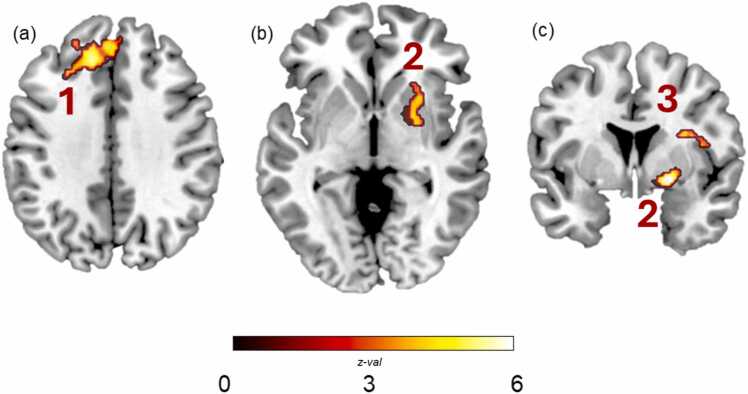
Activity for the arousal parametric modulator during the Passage Reading condition. Positive activity indicated that as arousal increased, so too did activity in the following regions: (a) left dmPFC (cluster 1), (b & c) right putamen and amygdala (cluster 2), and (c) right posterior insula (cluster 3). TFCE results displayed at *p* FDR-corrected < .05. *Note*: Image 4a is shown at slice X: −12, Y: 36, Z: 36 and images in 4b and 4c are shown at slice X: 26, Y: 2, Z: −4.

Next, we investigated which brain regions showed BOLD activity that increased or decreased in time with the intensity of the maximum arousal parametric modulator during passage reading (Fig. 4; Table 4). As idea unit arousal increased, activity in the right posterior insula, putamen and amygdala, and left dorsomedial PFC (dmPFC) also tended to increase. These findings are largely in alignment with the adult literature on neural responsiveness to arousal during reading, with core regions of situation model building and affect processing implicated here.

**Table 4:** Activations for the arousal parametric modulator during Passage Reading

Cluster #	Cluster size (*k*)	Anatomical region	BA	Max *T*	x	y	z
1	231	LH dmPFC LH dmPFC RH dmPFC	32 32 9	4.74 4.63 4.21	-13 -1 1	34 42 36	36 34 42
2	197	RH putamen RH amygdala parahippocampal gyrus RH anterior insula	— 34 48	5.54 4.27 4.18	21 22 23	4 -6 22	-8 -9 0
3	215	RH precentral gyrus RH posterior insula	48 48	5.15 4.97	47 43	-10 -7	22 15

Table 4. Note: Cluster size in mm^3^. TFCE results reported at *p* FDR-corrected < .05. BA: Brodmann area, dmPFC: dorsomedial prefrontal cortex.

### 3.3 Association of comprehension with arousal responsiveness during Passage Reading

**Fig. 5:**
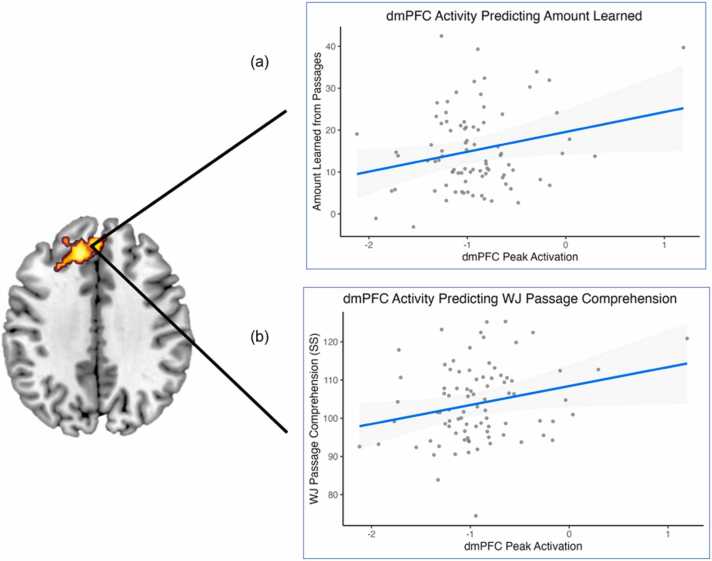
Partial regression plots showing that peak activation within the left dmPFC was associated with (a) amount learned (comprehended) from the in-scanner passages and (b) an out-of-scanner measure of general reading comprehension ability (WJ PC).

After confirming the effect of arousal on neural activity during passage reading, we examined the relationship between neural responsiveness to arousal by predicting: 1) the amount learned from reading the in-scanner passages and 2) a general reading comprehension measure (WJ PC). To capture individual phrase arousal responsiveness, we extracted peak activations for each subject from the three clusters that showed a positive association between phrase-level arousal and activity: right posterior insula, right amygdala/putamen, and left dmPFC. After extracting the three peaks for each subject, we ran two hierarchical regressions per peak value using the linear regression function in IBM SPSS Statistics for Windows, Version 31.0 (IBM, 2024): one predicting the amount learned while reading the in-scanner passages and another predicting WJ PC. This resulted in six sets of hierarchical regressions in total, two per peak. For each regression, we examined whether *R*^2^ change was significant across three steps: (1) age, gender, SES, WJ Word Attack; (2) the peak activation value, and (3) the interaction between the peak value and WJ Word Attack. To account for multiple comparisons across outcomes, a false discovery rate (FDR) correction (Benjamini-Hochberg) was applied to the *p*-values corresponding to the incremental model fit at step 1 and change in model fit at steps 2 and 3. Of note, inclusion of Word Attack scores (and their interactions) allowed us to examine whether basic word reading ability modulated how peak arousal activity impacted amount learned in-scanner and/or predicted general reading comprehension ability (WJ PC). Word Attack scores were chosen as the measure of reader ability because they measure phonological decoding skills—one of the most important predictors of reading ability during third grade (Foorman, Petscher, and Herrera, 2018)—and because previous evidence indicates that real word reading (versus non-word reading) is more highly correlated with SES (Romeo et al., 2018; van der Kleij et al., 2023). The results, by peak, are reported below:

dmPFC: For the regression predicting amount learned in the scanner: age, gender, SES and Word Attack explained 12.1% of the variance in the amount learned in the scanner (*R*^2^ = 0.121, *F*_4,81_ = 2.78, *p* = .032; FDR-corrected *p* = .032; age: *β* = 0.07, *t* = 0.65, *p* = .521, gender: *β* = 0.20, *t* = 1.81, *p* = .074, SES: *β* = 0.17, *t* = 1.57, *p* = .120, Word Attack: *β* = 0.26, *t* = 2.33, *p* = .022). These results indicate that after accounting for control variables, higher decoding ability was associated with more learning of the in-scanner passages. At step 2, the addition of left dmPFC peak activity in the model further explained 4.6% of the variance in amount learned, representing a significant improvement in model fit (Δ*R*^2^ = 0.046, Δ*F*_1,80_ = 4.46, *p* = .038; FDR-corrected *p* = .038). Peak dmPFC activity was positively associated with children’s learning of the in-scanner passages (*β* = 0.23, *t* = 2.11, *p* = .038). Hence, children who had higher activity in the left dmPFC when reading passages, and thus higher neural responsiveness when reading emotionally salient stimuli, learned more from those same passages (Fig. 5a). For step 3, the addition of the interaction between Word Attack and dmPFC peak did not explain any additional variance (Δ*R*^2^ = 0.009, Δ*F*_1,79_ = 0.89, *p* = .349; FDR-corrected *p* = .423) and the interaction term did not reach significance (*β* = 1.12, *t* = 0.94, *p* = .349), suggesting that arousal enhances reading comprehension for all readers, regardless of decoding ability.

For the regression predicting WJ PC: age, gender, SES and Word Attack explained 29.5% of the variance (*R*^2^ = 0.295, *F*_4,81_= 8.48, *p* < .001; FDR-corrected *p* = .002; age: *β* = −0.19, *t* = -1.97, *p* = .052, gender: *β* = −0.08, *t* = -0.83, *p* = .407, SES: *β* = 0.32, *t* = 3.39, *p* = .001, Word Attack: *β* = 0.31, *t* = 3.09, *p* = .003). These results indicate that, as expected, after accounting for age, gender, and SES, higher decoding ability was associated with higher WJ PC. Additionally, SES was positive associated with WJ PC. At step 2, the addition of left dmPFC peak activity in the model further explained 3.7% of the variance in WJ PC, representing a significant improvement in model fit (Δ*R*^2^ = 0.037, Δ*F*_1,80_ = 4.49, *p* = .037; FDR-corrected *p* = .038). This model reveals that children with stronger neural responsiveness to arousal fluctuations during passage reading tended to have higher WJ PC scores (*β* = 0.20, *t* = 2.12, *p* = .037; Fig. 5b). As was the case with amount learned, the addition of the interaction between Word Attack and dmPFC peak in step 3 did not explain any additional variance (Δ*R*^2^ = 0.005, Δ*F*_1,79_ = 0.65, *p* = .423; FDR-corrected *p* = .423) and the interaction term did not reach significance (*β* = −0.86, *t* = -0.81, *p* = .423).

Together, these results suggest a possible unique role of the dmPFC in situation model building. Additionally, arousal may enhance this situation model building, and subsequently, reading comprehension—across the spectrum of basic reading (decoding) abilities.

Amygdala: After FDR correction, neither regression reached statistical significance.

Insula: After FDR correction, neither regression reached statistical significance.

## 4 Discussion

The present study examined how fluctuations in phrase-level arousal modulate neural responses during passage reading in children. Neurocognitive models have long established that reading engages a distributed network of brain regions involved in language, vision, executive functioning, memory, and emotion (Bailey et al., 2018, Landi et al., 2013, Schlaggar and McCandliss, 2007). However, arousal, or the intensity of emotional activation elicited by a stimulus (Bradley and Lang, 1999; Russell, 1980), is largely underexplored in relation to the high-level reading network. Prior research has shown that emotionally salient passages modulate activity in regions such as the medial PFC and amygdala (Ferstl et al., 2005, Hsu et al., 2015, Wallentin et al., 2011)—structures involved in both general emotion processing and situation model formation (Yang et al., 2019). However, these findings to date have been limited to adults and have assessed passage arousal only at the global level. The current investigation fills this gap by examining how phrase-level arousal during passage reading influences brain activation in developing readers. Further, it explores whether neural responsiveness to arousal is modulated by a measure of reader ability. Grounded in theoretical frameworks such as the Construction-Integration Model (Kintsch, 1988), the Reading Systems Framework (Perfetti, 1999), and the Neurocognitive Poetics Model of Literary Reading (Jacobs, 2011, 2015), we examined whether the manipulation of local emotional salience is associated with situation model formation via neural activity and measures of reading comprehension. Indeed, emotionally arousing words have been shown to enhance lexical processing (Citron et al., 2014, Recio et al., 2014), and arousal at the word-level has been associated with faster recognition and increased neural activity in emotion-related regions (Hamann and Mao, 2002, Lewis et al., 2007, Straube et al., 2011). Despite this theoretical and empirical groundwork, studies have yet to link local-level arousal to neural activity in a cohort of younger readers or examine its relation to reading comprehension outcomes. Our robust sample of third-grade students read two passages during fMRI scanning. We quantified phrase-level arousal using norms from Warriner and colleagues (2013) to investigate how these ratings influenced whole-brain neural activity using a parametric modulation analysis.

After ensuring that our novel mixed block/event-related design paradigm recruited expected reading regions (bilateral superior temporal sulci, anterior temporal lobes, medial prefrontal cortices, and visual cortices, and left angular gyrus, inferior frontal gyrus, precentral gyrus, insula, and vOTC; Aboud et al., 2016; Bailey et al., 2018; Landi et al., 2013; Schlaggar and McCandliss, 2007), the emotional content of each stimulus was quantified. Specifically, each passage was broken into smaller “idea units,” which were short, meaningful segments of text between 1 and 5 words. These idea units (or phrases) were used as stimuli. To quantify text arousal, all content words within a phrase were matched to arousal ratings from the Warriner database (2013) and the maximum arousal rating per phrase was used as the trial-level arousal value. Parametric modulation analysis with these ratings was used to examine which brain regions revealed BOLD fluctuations during passage reading that aligned with idea unit arousal fluctuations. Regions sensitive to arousal fluctuations were the right amygdala/putamen and posterior insula, and left dmPFC—largely in alignment with previous literature on emotion and salience processing in the brain (Beissner et al., 2013). More specifically, the amygdala and insula have direct connections to the literature on basic emotional processing and emotional discourse comprehension, whereas the putamen and dmPFC have connections to related literature on cognitive emotional processing. The amygdala, a subcortical limbic region, has consistently been implicated in processing highly arousing emotional stimuli both generally (Lindquist et al., 2012, Palomero-Gallagher and Amunts, 2022, Sabatinelli et al., 2005, Wager et al., 2003) and during word and passage paradigms investigating lexical arousal (Citron, 2012, Ferstl et al., 2005, Hamann and Mao, 2002, Hsu et al., 2015, Lewis et al., 2007, Straube et al., 2011, Wallentin et al., 2011). Similarly, the insula, located within the lateral sulcus, is active in studies of motivational states (Craig et al., 1998) and valenced stimuli (Viinikainen et al., 2010). The posterior insula, more specifically, reflects the integration of emotional and sensory experiences (Pessoa, 2017). Additionally, it is responsive to the affective values of words (Citron et al., 2014) and the range of lexical arousal ratings across a passage (Hsu et al., 2015). The putamen, another subcortical structure, is not typically thought of as a core affect processing region but has been associated with neural responsiveness during cognitive-emotional processing (Pierce and Péron, 2020). Similarly, the dmPFC is conceptualized as a regulatory region responsible for connecting lower-level emotional processing with higher-level cognitive processes (Etkin et al., 2011); recent research has also posited these processes may contribute to situation model building during reading (Buckner et al., 2008, Mar, 2011, Simony et al., 2016).

Critically, in our investigation, we found that peak activity within the dmPFC for the arousal parametric modulation analysis during passage reading was associated with two measures of reading comprehension ability; amount learned during passage reading and a general reading comprehension measure (WJ PC). While linked to response to emotional stimuli (Phan et al., 2004), the dmPFC is considered a situation model processing region (Ferstl et al., 2005) and a core DMN region (Raichle, 2015). The region of activity from our analysis shows topographical concordance with a meta-analysis on discourse processing (Yang et al., 2019), demonstrating that the dmPFC is a critical high-level cognition region that responds in time to textual passage information (here, arousal), and is linked to behavioral reading trajectories. We posit that the dmPFC may act as the link between emotional and reading brain regions, working at the crossroads between the two to result in coherent situation model formation and skilled reading. Thus, we hypothesize that higher activation to more arousing phrases during reading promotes better reading comprehension. Of note, however, our current study design does not allow us to disentangle the direction of the effect; it could be that better readers have higher activation to high-arousal phrases, which promotes better reading comprehension. Future causal work is needed to clarify this effect. We additionally hypothesized that amygdala responsiveness to arousal during reading may also have been predictive of reading outcomes but did not see this relationship in our sample. While the amygdala has been implicated in discourse processing when arousal is measured at the passage-level (Hsu et al., 2015), it is possible that this subcortical structure is less fine-tuned to granular fluctuations in arousal and responds to general passage emotional states, making it less predictive of reading outcomes.

Taken together, this parametric modulation analysis revealed that both lower- and higher-level emotion processing regions in the brain modulated with phrase-level arousal fluctuations during passage reading. Further, these fluctuations within the dmPFC specifically were associated with higher reading comprehension both in and out of the scanner. We assert that these cortical arousal processing regions, and particularly the dmPFC, may coordinate with reading and language regions to improve situation model development and give rise to a coherent mental percept that aids in comprehension and learning, though further research is needed to support this claim. Regardless, children demonstrated the involvement of both bottom-up and top-down neural circuitry critical for reading and emotion, as was seen previously in adults. Importantly, this study is the first of our knowledge to examine how phrase-level arousal influences brain activity in a pediatric population during a passage reading task, thus bridging a critical gap in the literature that has focused predominantly on adults and global (i.e., passage-level) emotional content. This distinction is crucial, as it suggests that children’s neural situation model systems are sensitive to emotional shifts embedded within fine-grained units of text. Because the effect of salient emotion on neural activity and reading comprehension did not differ based on children’s decoding ability, these results suggest emotional manipulation of texts could be leveraged to support reading comprehension development for all readers; however, additional research within a targeted sample of struggling readers may be needed to confirm these initial findings. Finally, because engagement of the dmPFC is also linked to situation model building and comprehension in adults (e.g., Simony et al., 2016; Yarkoni et al., 2008), we hypothesize that adults with varying reading abilities would show similar associations of arousal responsiveness in the dmPFC to reading comprehension.

### 4.1 Limitations and future directions

While the current study provides further insight into the neurocognitive underpinnings of emotional discourse comprehension, it will be critical to extend this study with a specific cohort of struggling readers, as well as with a more diverse sample, to understand whether the (potential) facilitatory role of embedding higher-arousal words in text could be utilized to enhance comprehension. Direct manipulation of textual arousal ratings may be especially fruitful for probing this possibility. Additionally, because our analyses focused on the arousal ratings of constituent words within the passages, rather than self-ratings of arousal at the passage-level, we could not compare local- and global-level arousal metrics. However, our methods provide a more objective measurement of emotional fluctuations in the texts, and we draw on the study by Hsu and colleagues (2015), which showed considerable neural overlap between whole-passage affective ratings by individuals and arousal means derived from rating constituent words. Also of note, the current study examined emotional salience and its impact on reading cognition by measuring phrase arousal and did not include measures of valence. We were unable to extend the research question to include valence because the passages did not have high valence variability. While these passages were not created specifically for this intended research purpose, subsequent studies that we have conducted have revealed that valence can be difficult to manipulate with child participants due to Institutional Review Board concerns. Thus, while these results suggest that these fluctuations of neural regions may also occur in response to changes in valence, future research (which may be confined to adult participants) is necessary to corroborate this interpretation. Lastly, to further understand the cascading nature of emotional discourse processing in the brain, functional effective connectivity analyses would be critical and informative for the field at large. This is especially true of future research aiming to extend this work by directly measuring the dynamic fluctuations in neural responsiveness to emotionally manipulated stimuli, rather than using peak activity. Such research could further examine how these individual differences relate to reading comprehension; for example, whether variability in moment-to-moment responsiveness of the dmPFC during reading differentiates good versus poor readers.

### 4.2 Implications

These findings contribute to our growing understanding of how textual arousal influences reading and, importantly, extend this work to a sample of young readers. From a theoretical perspective, these results underscore the need to more fully integrate affective components into cognitive models of reading. This work may also have meaningful implications for educational practice, pointing to the potential benefits of incorporating emotionally engaging text features into reading instruction to support comprehension in developing readers of all abilities. Future work should investigate whether such approaches are especially beneficial for readers that have both oral language comprehension and decoding deficits, as this preliminary work suggests boosting engagement through emotional resonance may help strengthen comprehension pathways. In sum, this study lays critical groundwork for future neuroimaging research aimed at unpacking further mechanisms by which arousal influences reading comprehension, with the long-term goal of informing targeted, evidence-based interventions.

## CRediT authorship contribution statement

**Laurie E. Cutting:** Writing – review & editing, Writing – original draft, Supervision, Funding acquisition, Data curation, Conceptualization. **Sarah S. Hughes-Berheim:** Writing – review & editing, Data curation, Conceptualization. **Andrea N. Burgess:** Writing – review & editing, Writing – original draft, Visualization, Formal analysis, Data curation, Conceptualization.

## Declaration of Competing Interest

The authors declare that they have no known competing financial interests or personal relationships that could have appeared to influence the work reported in this paper.

## Data Availability

Data will be made available on request.
